# Commentary: Recommendation of Antimicrobial Dosing Optimization During Continuous Renal Replacement Therapy

**DOI:** 10.3389/fphar.2020.580163

**Published:** 2020-09-16

**Authors:** Elodie Matusik

**Affiliations:** ^1^ Department of Pharmacy, Valenciennes General Hospital, Valenciennes, France; ^2^ Department of Intensive Care Research, Valenciennes General Hospital, Valenciennes, France

**Keywords:** anti-infective agents, antimicrobials, pharmacokinetics, pharmacodynamics, critical care, continuous renal replacement therapy, therapeutic drug monitoring, dosing optimization****

## Introduction

Facing challenges of appropriate antimicrobial dosing during Continuous Renal Replacement Therapy (CRRT), Li et al. recently published a review to optimize efficacy and to limit toxicity (Li et al., 2020). Although it is helpful for clinician at patient bedside to choose dosing in the absence of pharmacokinetic individual data, some limitations should be highlighted. One dose does not fit all and antimicrobial optimization in critically ill patients requires a holistic approach applying different pharmacokinetic-pharmacodynamic (PK-PD) principles not only based on CRRT modalities.

## Consider Inter and Intra-Individual Variability

Existing literature presents many limitations. As noticed by Li et al., CRRT practices are heterogeneous leading to subsequent diversity in antimicrobial dosing (Churchwell and Mueller, 2009; Lewis and Mueller, 2014). Other authors demonstrated low quality of CRRT pharmacokinetic studies and lack of key information in the literature needed to define dosing regimens, particularly the delivered CRRT dose (Li et al., 2009; Vaara et al., 2012). The studies often only include a few patients, unrepresentative of the population with single or multi-compartmental models. Critical care populations variety may jeopardize the extrapolation of the pharmacokinetic results (Ulldemolins et al., 2014). Pathophysiology of the diseases and patients severity are different leading to wide pharmacokinetics variations, as described in the DALI study reporting that beta-lactams concentrations could vary by a factor of 100 from patient to patient in Intensive Care Unit (ICU) (Roberts et al., 2014). Therefore, pharmacokinetic parameters of healthy volunteers cannot be used.

Extreme inter/intra-individual pharmacokinetic variability in ICU and potential PK-PD uncertainties necessitate dosing simulations in different clinical contexts to suggest appropriate dosing regimens at individual level (Dhaese et al., 2019). As noticed by Li, his recommendations may be not sufficient for patients with residual renal function. Dynamic change of patient’s clinical status with subsequent modifications of pharmacokinetics should be considered for dosing adaptation (Choi et al., 2009; Guilhaumou et al., 2019). Underdosing is over-prevalent during the initial septic phase and antimicrobial initial doses should consider the increase of volume of distribution (De Waele and Carlier, 2014; Wong et al., 2015). However, this requires sophisticated PK-PD models integrating patients and population data. Subsequent doses should be decided according to total clearance. Therefore, fixed doses do not appear appropriate during CRRT.

## Consider All Pharmacokinetic Changes to Define Dose Adjustment

As noted by Li, drug clearance depends on CRRT modalities. Potential drug infusion incompatibilities and interactions with the whole circuit should also be consider, including tubing, especially when polyvinylchloride (PVC) is used (Preston et al., 2007; Shekar et al., 2015). Lack of CRRT standardization leads to high pharmacokinetics differences requiring *in vitro* data to study the impact of CRRT modalities.

Other pharmacokinetic modifications than CRRT may impact antimicrobial concentrations, as fluid balance variability, protein binding modifications and hepatic function. This may impact the PK-PD target attainment (Ulldemolins et al., 2011; Lewis and Mueller, 2014; Vanstraelen et al., 2014; Kurland et al., 2019). Some studies consider total antimicrobial concentration instead of active unbound concentration without possible interpretation of the target attainment (Roberts et al., 2013). Precaution should therefore be taken to define dosing regimens.

## Consider PK-PD Targets and Infusion Mode

Li did not clearly describe PK-PD targets selected to define antimicrobial dosing. Lack of consensus for some antimicrobials as beta-lactams leads to different dosing regimens recommendations, as reported for meropenem (PK-PD target from 40% T > MIC to 5 x 100% T > MIC) (Kawano et al., 2015; Ulldemolins et al., 2015). PK-PD targets and subsequent dosing depend on bacteria sensibility and should be consider for all antimicrobials.

Infusion mode is crucial to guide dosing regimens, optimizing the probability of PK-PD target attainment, limiting potential toxicity and the emergence of bacterial resistance. The randomized controlled BLISS study demonstrated higher clinical cure rates in septic patients with continuous infusions of beta-lactams than with intermittent infusions for the same daily dose, and higher PK-PD target attainment rates at 100% *f*T > MIC (Abdul-Aziz et al., 2016). However, extended and continuous infusions should not be used without protocolization to assure stability, especially for carbapenems and amoxicillin+/-clavulanic acid. Continuous/extended infusions including a loading dose were therefore recommended for beta-lactams in some clinically contexts or in case of severity (Guilhaumou et al., 2019). Moreover, the same intermittent infusions dosing cannot be applied to continuous and extended infusions to obtain a defined target concentration. When PK-PD target is attained, continuous/extended infusions also allow a daily dose reduction permitting to limit toxicity (Guilhaumou et al., 2019).

## Consider Therapeutic Drug Monitoring (TDM) and PK-PD Models

Regarding inter/intra-individual variability, individualized PK-PD targets and dosing are required (Goldstein and Nolin, 2014; Roberts and Roberts, 2014; Shaw et al., 2016). Some European learnt societies recommended TDM as a standard of care for most antimicrobials in ICU, especially for patients treated by CRRT, and Li suggested it to optimize therapy (Guilhaumou et al., 2019; Abdul-Aziz et al., 2020). TDM allows a higher probability of PK-PD target attainment and to limit over and underdosing, especially when Monte Carlo simulations are not available in the population of interest. To assure better accuracy of dose adaptations, a modeling population pharmacokinetic approach should be used, permitting to consider inter/intraindividual variations (Fuchs, 2015; Penetrat-Roger, 2018). Bayesian approach, established on conditional probabilities, permits to predict individual antibiotic concentrations integrating data from population models and individual pharmacokinetic parameters, bacterial ecology and previous concentrations observed.

Many factors are required to determine appropriated dosing regimens for patients treated by CRRT. Dosing data from the literature can be applied under the strict similar conditions of the studies (Choi et al., 2009). Facing pharmacokinetic variability in critically ill patients, CRRT practices heterogeneity and the bacterial local ecology, TDM and Bayesian modelling represent the best options to optimize antimicrobial dosing and their development should be a priority to individualize dosing. However, in the absence of sophisticated pharmacokinetic tools, Li’s recommendations contribute to give an order of magnitude of CRRT impact and may help to avoid underdoses/overdoses on the basis of the available data, with a possible integration in an antibiotic stewardship program considering other PK-PD considerations.

## Author Contributions

The author confirms being the sole contributor of this work and has approved it for publication.

## Conflict of Interest

The author declares that the research was conducted in the absence of any commercial or financial relationships that could be construed as a potential conflict of interest.

## Abbreviations

CRRT, Continuous Renal Replacement Therapy; ICU, Intensive Care Unit; PK-PD, Pharmacokinetic-Pharmacodynamic; TDM, Therapeutic Drug Monitoring.
